# Undifferentiated Pleomorphic Sarcoma of the Thoracic Aorta: A Diagnostic and Therapeutic Challenge

**DOI:** 10.1016/j.atssr.2023.02.002

**Published:** 2023-02-20

**Authors:** Brian M. Till, Amit Java, Colin King, Vishal N. Shah, Baskaran Sundaram, Wei Jiang, Michael J. Nooromid, Konstadinos A. Plestis

**Affiliations:** 1 Department of Surgery, Thomas Jefferson University Hospital, Philadelphia, Pennsylvania; 2 Department of Surgery, University of Nebraska Medical Center, Omaha, Nebraska; 3 Department of Radiology, Thomas Jefferson University Hospital, Philadelphia, Pennsylvania; 4 Department of Pathology, Thomas Jefferson University Hospital, Philadelphia, Pennsylvania

## Abstract

We describe the workup and treatment of a 52-year-old man presenting with an extensive primary tumor of the descending aorta. Workup before surgical resection suggested nonneoplastic causes of this lesion, and we detail the diagnostic and therapeutic challenge posed by these rare tumors.

Primary tumors of the great vessels are exceedingly rare conditions typically characterized by late diagnosis and poor survival. Both the identification and treatment of these lesions can pose considerable challenges. We describe the use of multiple imaging modalities to characterize an aortic lesion and its surgical resection. The patient has recovered uneventfully and is now undergoing adjuvant treatment.

A 52-year-old man presented with 3 months of lower back pain worsening with activity and intermittent paresthesia in the right lower extremity. He denied claudication. His past medical history was notable for essential hypertension. His physical examination and laboratory findings were unremarkable. Computed tomography angiography (CTA) of the aorta demonstrated a large, irregular, low-attenuation intraluminal filling defect in the descending aorta with an outpouching of the anterior aspect of the descending thoracic aorta measuring 2.7 × 3.1 cm. This outpouching demonstrated central low attenuation and rim enhancement (Figure 1). The lesion caused severe luminal narrowing. In addition, there was wall thickening, focal dilation, and thrombosis of the proximal superior mesenteric artery (SMA) and small cortical infarcts in both kidneys. The patient underwent gadolinium-enhanced multiphasic magnetic resonance angiography (MRA; Figure 2). This examination confirmed the noncontiguous aortic and SMA findings and demonstrated wall thickening and hyperenhancement throughout the wall of the descending thoracic aorta with nonenhancing filling defect in the anterior outpouching of the descending aorta. The differential diagnosis included pseudoaneurysm associated with extensive thrombus secondary to vasculitis and aortic tumor (with pseudoaneurysm due to direct extension into the mediastinum). Given the possibility of esophageal encroachment, esophagoscopy with endoscopic ultrasound was performed and showed a 2.8 × 1.5-cm hypoechoic area adjacent to the aorta with no esophageal encroachment.

**Figure 1:**
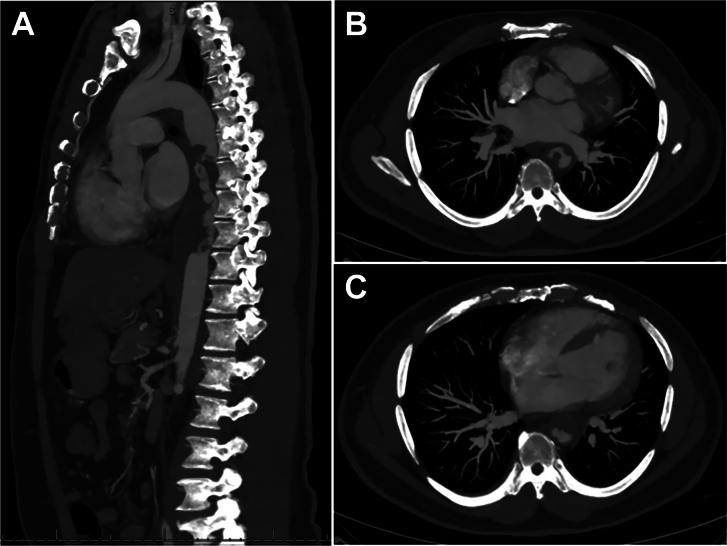
(A) Computed tomography angiogram (CTA) in sagittal plane. (B) Axial CTA. (C) Axial CTA.

**Figure 2:**
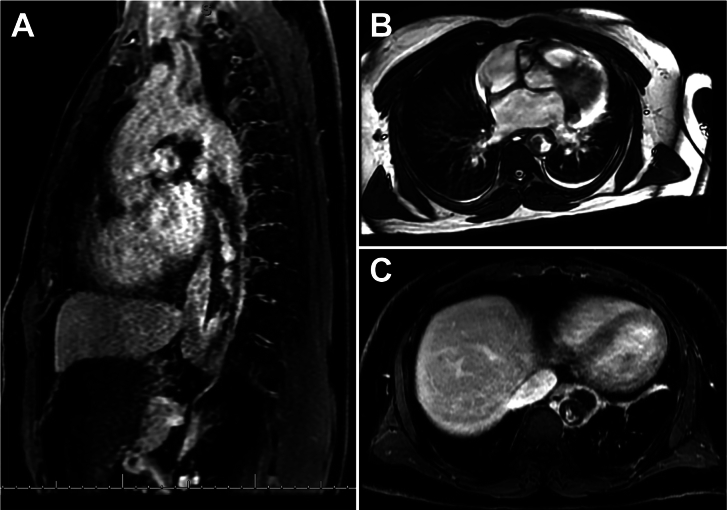
(A) Magnetic resonance angiogram (MRA) in sagittal plane after contrast enhancement. (B) Axial MRA, T1-weighted image before contrast enhancement. (C) Axial MRA, T1-weighted image after contrast enhancement.

The patient was brought to the operating room and intubated with a double-lumen endotracheal tube. Continuous neuromonitoring with somatosensory evoked potentials and motor evoked potentials and cerebrospinal fluid drainage were established. A thoracoabdominal incision in the sixth intercostal space was used. The descending thoracic aorta was completely mobilized. Because of severe intraluminal narrowing, the aortic arch and abdominal aorta were cannulated. The left common femoral vein was cannulated. The patient was placed on cardiopulmonary bypass and cooled to 22°C. Circulatory arrest was initiated, and the aorta was transected 1 cm below the left subclavian and proximal to the celiac axis. After the proximal anastomosis was completed, the graft was deaired and antegrade perfusion was initiated. After distal anastomosis, full cardiopulmonary bypass was established and the patient was rewarmed. The patient came off bypass without difficulty. He had an uneventful postoperative course. Subsequent positron emission tomography scan demonstrated a hypermetabolic mesenteric lymph node and impending fracture of the left femur due to mid-diaphyseal metastasis. He underwent prophylactic left femur fixation. The patient was prescribed doxorubicin and ifosfamide chemotherapy.

Final pathologic examination revealed high-grade undifferentiated pleomorphic sarcoma with extensive necrosis. The tumor measured 17 cm in greatest dimension and showed high-grade spindle and pleomorphic sarcoma, with 49 mitoses per 10 high-power fields (Figure 3). Both proximal and distal margins showed microscopic foci of tumor. Intimal sarcoma was the leading differential diagnosis; however, fluorescence in situ hybridization was negative for *MDM2* gene amplification. Negative α-smooth muscle actin and desmin immunostains ruled out a leiomyosarcoma, and negative endothelial markers ERG and CD31 ruled out angiosarcoma. The tumor was therefore classified as undifferentiated pleomorphic sarcoma.

**Figure 3:**
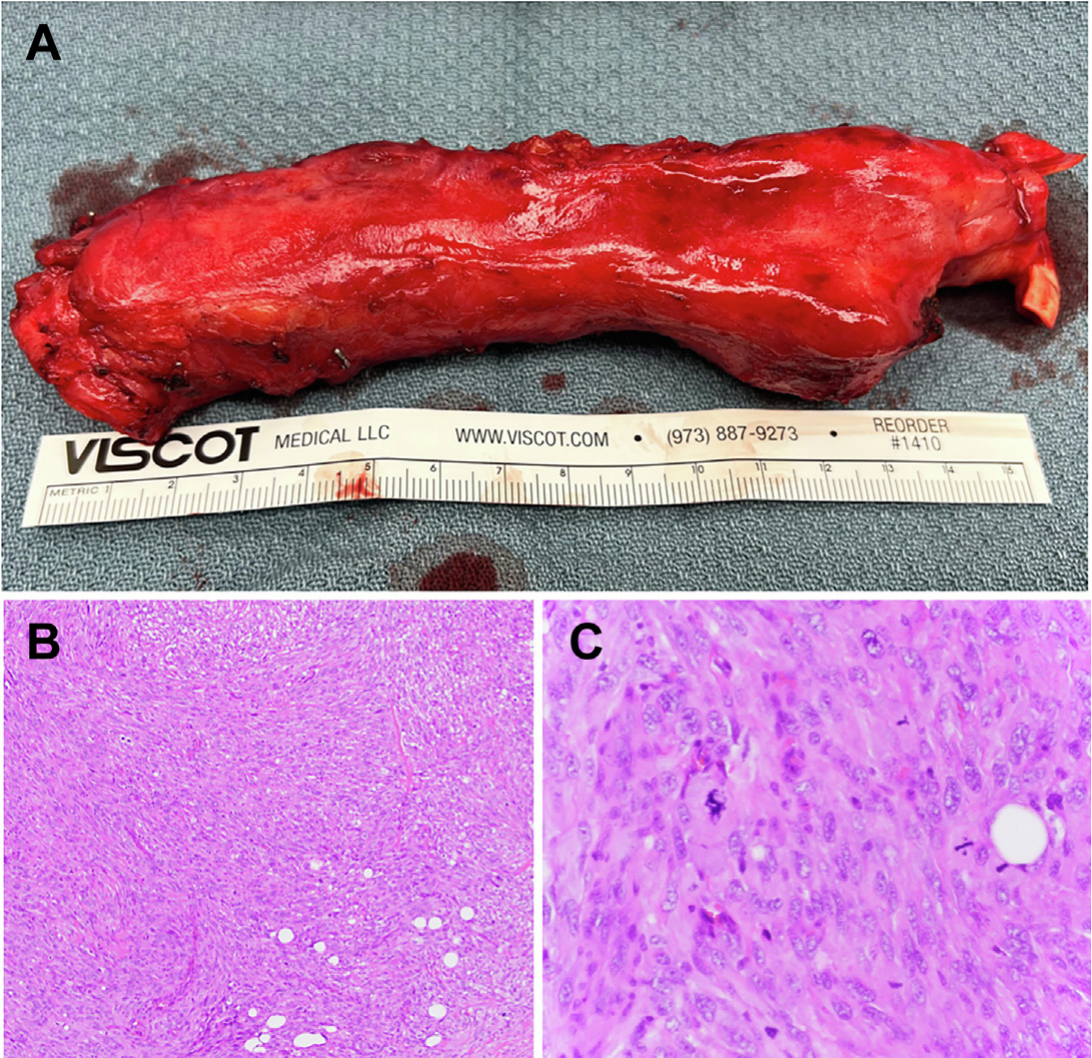
(A) Gross surgical specimen. (B) Histologic slide, hematoxylin and eosin stain at low power. (C) Histologic slide, hematoxylin and eosin stain at high power.

## Comment

Sarcomatous tumors of the aorta pose considerable diagnostic and therapeutic challenges. Based on CTA, the extent of the lesion and severe luminal narrowing were identified, but definitive diagnosis could not be established. For this reason, MRA was obtained to better characterize the soft tissue involvement. In these studies, the lesion lacked the degree of T1 enhancement suggestive of an angiosarcoma or other malignant lesions. The large size of the lesion, diffuse wall thickening, and noncontiguous involvement of the SMA further suggested a vasculitis rather than a primary malignant neoplasm. Given near-obstruction of the lumen and the threat of visceral ischemia, the decision was made to proceed with operative intervention.

Approximately 200 primary sarcomatous tumors of the aorta have been reported between 1873 and 2017, with a large proportion of cases found at time of postmortem examination.^1^ Tumors show a male predominance and a mean age at presentation of 60.1 years.^2^ Lesions are predominantly found in the abdominal aorta and are less frequently observed in the thoracic aorta or arch. They are subclassified according to cell of origin; undifferentiated sarcoma is the most common subtype, followed by angiosarcoma, intimal sarcoma, and leiomyosarcoma.^2^ Patients typically present with advanced disease, and chief complaints are most frequently related to peripheral or visceral embolization.^3^^,^^4^

Aortic sarcomas are characterized by dismal prognosis. Approximately half of patients are found to have metastasis at time of presentation.^5^ Median survival at diagnosis is 8 months, and estimates of 5-year survival range from 3.5% to 8.8%.^2^^,^^5^ No relationship has been established between the segment of aortic involvement and survival, nor has a relationship between histologic subtype and survival been identified.

Sarcomatous aortic lesions are characterized by low sensitivity to neoadjuvant therapy,^3^ and R0 resection is recognized as critical for extended survival. In this case, despite 2 cm of gross margins, the final pathologic examination revealed positive microscopic margins. The use of palliative thoracic endovascular stent was considered in this case but deemed impossible, given the degree of luminal narrowing and the large tumor burden. The two largest retrospective case series of aortic sarcoma have each identified significant median survival benefit for those treated with adjuvant therapy.^2^^,^^5^ Doxorubicin-based regimens are favored, and this patient is scheduled for 6 cycles of doxorubicin and ifosfamide therapy based on EROTC 62012.

In conclusion, we present the case of an extensive thoracic aortic undifferentiated pleomorphic sarcoma manifested with back pain and radiologic evidence of embolic disease. This case demonstrates the diagnostic and therapeutic challenges posed by these tumors. Whereas CTA often plays a critical role in identifying lesions, the ability to differentiate tumor from other disease is limited in this medium. MRA is considered the “gold standard” in diagnosis, but aortic sarcomas characterized by large necrotic burden can pose a considerable diagnostic challenge despite use of advanced imaging modalities.

## References

[1] Shuster T.A., Dall'Olmo C.A., Spadone D., Silver D. (2002). Abdominal aortic sarcoma: report of a case with long-term survival and review of the literature. Ann Vasc Surg.

[2] Vacirca A., Faggioli G., Pini R. (2020). Predictors of survival in malignant aortic tumors. J Vasc Surg.

[3] Salhab K.F., Said S.M., Sundt T.M. (2012). Pseudocoarctation of the aorta secondary to aortic intimal sarcoma. Ann Thorac Surg.

[4] Seelig M.H., Klingler P.J., Oldenburg W.A., Blackshear J.L. (1998). Angiosarcoma of the aorta: report of a case and review of the literature. J Vasc Surg.

[5] Rusthoven C.G., Liu A.K., Bui M.M. (2014). Sarcomas of the aorta: a systematic review and pooled analysis of published reports. Ann Vasc Surg.

